# Adaptive fixed-time fuzzy containment control for uncertain nonlinear multiagent systems with unmeasurable states

**DOI:** 10.1038/s41598-024-66385-3

**Published:** 2024-07-09

**Authors:** Ruixia Liu, Lei Xing, Yongjian Zhong, Hong Deng, Weichao Zhong

**Affiliations:** 1 School of Automation, Xi’an University of Posts and Telecommunications, Xi’an, 710072 China; 2 Research Center of Satellite Technology, Harbin Institute of Technology, Harbin, 150001 China; 3 Shanghai Electro-Mechanical Engineering Institute, Shanghai, 201109 China; 4 Shanghai Institute of Satellite Engineering, Shanghai, 201109 China

**Keywords:** Containment control, Adaptive backstepping control, State observer, Fixed-time control, Engineering, Mathematics and computing

## Abstract

This paper addresses the adaptive fixed-time fuzzy containment control for uncertain nonlinear multiagent systems, where the states and nonlinear functions are not feasible for the controller design. To address the issue of unmeasurable states, a state observer is developed, and fuzzy logic systems are utilized to approximate unknown nonlinear functions. Under the framework of fixed-time Lyapunov function theory and cooperative control, an adaptive fixed-time fuzzy containment control protocol is derived via the adaptive backstepping and adding one power integrator method. The derived fixed-time containment controller can confirm that the closed-loop systems are practical fixed-time stable, which implies that all signals in the systems are bounded and all follower agents can converge to the convex hull formed by the leader agents within fixed-time in the presence of unmeasurable states and unknown nonlinear functions . Simulation examples are conducted to test the validity of the present control algorithm.

## Introduction

For several decades, containment control for multiagent systems (MASs) as a persistent research area in cooperative control that has found applications in various kinds of engineering systems such as Earth monitoring, semi-Markovian MASs, obstacle avoidance of robots, and autonomous underwater vehicle systems^1–5^. The primary aim of implementing containment control is to guarantee that the derived control protocol enables all follower agents to converge to the convex hull formed by the leader agents. Since the containment controller design problem of the linear MASs with both dynamic and stationary leader agents was solved by Cao et al.^6^, rich containment works for different linear systems have been presented^7,8^. A formation-containment issue was investigated by Wang et al.^9^ for interacted heterogeneous linear systems by fusing impulsive control method and discrete-time communication manner in the distributed hybrid active control scheme design. In reality, most practical systems are subject to complex nonlinear characteristics and should be modeled as uncertain nonlinear systems^10^. Because of the ever-increasing popularity of approximate techniques, especially in fuzzy logic systems (FLSs)^11–13^, more and more containment control algorithms, together with the backstepping technique, are executed over uncertain nonlinear MASs^1,6^.By utilizing fuzzy state observers and backstepping technique, a containment controller was formulated by Zhang et al.^14^ for MASs in the nested pure-feedback non-affine nonlinear structure. The adaptive distributed containment control law was derived by constructing FLSs, which ensured the containment error is bounded of the nonlinear strict-feedback systems by Wang et al.^15^. However, the containment controllers designed in some references^1,6,14,15^ can only ensure the controlled systems are asymptotically stable or uniformly ultimately bounded.

For containment control problems, estimating controller performance relies heavily on the convergence rate, making it a critical criteria. Notice that the finite-time control methods have better disturbance-rejection capabilities, faster convergence speed, and higher tracking accuracy when compared to asymptotic controllers^16–21^. Hence, the works on the finite-time containment control issues for MASs is of great importance and a sets of valuable studies were presented in the references^22–24^. In the early days, a finite-time containment control strategy was developed in reference^25^ for rigid bodies by merging the sliding mode control approach, which also discusses the cases of dynamic leaders and a single stationary leader. In some references^26,27^, two distributed finite-time containment control strategies formulated to obtain fixed-time stability were presented for multiple unmanned aerial vehicles and double-integrator MASs. Further, to obtain ideal convergence rate for the controlled MASs, in reference^24^, the finite-time containment control framework was established with adding one power integrator for MASs with mismatched disturbances and the finite-time stability sufficient conditions have been given. More notably is that the needing of initial states to value the settling time is not conducive to the finite-time controllers utilization owing to the initial states are not always feasible in practical situations. Since this shortcoming, the fixed-time approach was derived by A. Polyakov^28^, where the settling time can be determined without the initial states. Due to the fixed-time controllers have distinct advantages, and as a result, significant progress has been made in the references^29–31^. By adding one power integrator approach, a fault tolerant adaptive fixed-time control was focused in some references^32^ by employing fuzzy logics systems, in which the unknown actuator faults were involved. In reference^33^, a fixed-time consensus control algorithm is derived via the sliding mode control approach, which is feasible for MASs with external disturbances.

In most of the aforementioned results are working on the constraint that all states are available to the control schemes propose. Unfortunately, in many engineering applications, such an assumption does not always hold. For example, due to cost-saving considerations, sensor failure, and load limitation, only the output signals of the systems are known^34–36^. One of the most effective approaches to this circumstance is to propose the output feedback control law^37^. Based on internal mode principle, a static output-feedback containment controller was derived for heterogenous MASs in reference^38^, while in reference^39^ an output finite-time containment controller was studied for heterogenous MASs consisting of both multiple followers and leaders. Note that the output feedback containment controllers have been formulated in some references^39–41^, but these results are only concerned with the asymptotically or finite-time stable. Although the fixed-time controller is derived in some references^32,42^, these algorithms may not be valid for the containment control. Moreover, when the unmeasurable states are incorporated, the fixed-time containment control protocol derive becomes more complicated. So far, the issue of developing fixed-time controllers for nonlinear uncertain systems with unmeasurable states has not been thoroughly investigated, especially for uncertain MASs containment control case. This research gap has prompted us to conduct this present study.

Summarizing the above discussions, an observer-based adaptive fixed-time fuzzy control protocol is formulated to handle the containment control of MASs with unmeasurable states and unknown nonlinear functions. The main contribution of this paper is threefold: (1) Different from the existing finite-time containment control algorithms, such as references^16,22^, a fixed-time adaptive fuzzy control protocol is derived, which the settling time of the resulting controlled MASs is regardless of initial conditions; (2) As the states of each follower cannot be directly measured, an observer-based fixed-time containment control protocol is formulated, which can confirm the controlled MASs are practical fixed-time stable and the observer errors are bounded within fixed-time; (3) In comparison with recent results on fixed-time control by using sliding mode control approach^33,43^, the developed fixed-time containment control protocol via the adaptive backstepping and adding one power integrator approach in this article can avoid the singular phenomenon. Although adaptive nonsingular fixed-time controllers have been addressed in some references^32,42^, the obtained results are not feasible for the containment control problem.

The rest of this paper is laid out as follows. In Sect. "Preliminaries and problem statement", the problem statement and key preliminaries are presented. In Sect. "State observer and containment control scheme design", the state observer and control protocol design are shown. The stability analysis for the uncertain MASs subject to unmeasurable states is studied in Section "Stability analysis". In Section "Simulation examples", simulation examples are shown. In Section "Conclusion", some conclusions are shown.

## Preliminaries and problem statement

### Graph theory

The algebraic graph theory below is employed to illustrate the communication of information among agents. The communication of information between the follower agents and the leader agents can be described by, $\mathfrak {G}=(\mathfrak {L},\pounds ,\mathfrak {A})$. $\mathfrak {L}=\{s_1,\dots ,s_N,s_{N+1},\dots ,s_{N+M}\}$ denotes the set of agents, where $i=1,\dots ,N$ denotes followers, $i=N+1,\dots , M+N$ denotes the leaders. $\pounds =\{(s_i,s_j)\}\in \mathfrak {L}\times \mathfrak {L}$ denotes the edge set. $\mathfrak {A}=[\alpha _{ij}]\in \mathbb {R}^{(N+M)\times (M+N)}$ denotes the adjacency matrix. $(s_i,s_j)\in \pounds$ denotes the agent *i* is a neighbor of agent *j*. $\alpha _{ij}>0$ for $(s_i,s_j)\in \pounds$; otherwise $\alpha _{ij}=0$. $\mathfrak {T}=[\mathfrak {T}_{ij}]\in \mathbb {R}^{(M+N)\times (M+N)}$ denotes the Laplacian matrix, and $\mathfrak {T}_{ij}=-\alpha _{ij}$ if $i\ne j$. The Laplacian matrix $\mathfrak {T}$ is,

1 $$\begin{aligned} \mathfrak {T}=\left[ \begin{array}{lll} \mathfrak {T}_1&{}\,\,\,\mathfrak {T}_2 \\ 0_{M\times N}&{}\,\,\,0_{M\times M} \end{array} \right] . \end{aligned}$$

where $$\mathfrak {T}_1\in \mathbb {R}^{M\times M}$$ and $$\mathfrak {T}_2\in \mathbb {R}^{M\times N}$$.

### Fuzzy-logic systems

The FLSs will be utilized to model the unknown nonlinear terms. The FLSs have the following property.

#### Lemma 1

Reference^44^ If *h*(*x*) is a continuous function on a compact set $\Pi$, for given $\varepsilon ^*>0$, there is the FLSs such that,

2 $$\begin{aligned} {\mathop {\textrm{sup}}\limits _{\textrm{x}\in \Pi }}|h(x)-\theta ^{*T}\varsigma (x)|\le \varepsilon ^*\end{aligned}$$

where $\varsigma (x)=[\varsigma _1(x),\varsigma _2(x),\dots ,\varsigma _g(x)]^T/\sum _{i=1}^g\varsigma _i(x)$ denotes the fuzzy basis faction vector, *g* represents the number of rules, $\theta ^{*}$ is the optimal parameter vector.

### System description

This article investigates the nonlinear multi-agent systems (MASs) that comprise of *M* leaders and *N* followers. The *i*th ($i=1,\dots ,N$) follower’s dynamic is represented by,

3 $$\begin{aligned} \left\{ \begin{array}{lll} \dot{x}_{i,1}=x_{i,2}+f_{i,1}(x_{i,1}), \\ \dot{x}_{i,m}=x_{i,m+1}+f_{i,m}({\bar{x}}_{i,m}),\,\,\,2\le m\le n-1\\ \dot{x}_{i,n}=f_{i,n}({\bar{x}}_{i,n})+u_i, \\ y_i=x_{i,1} \end{array} \right. \end{aligned}$$

where ${\bar{x}}_{i,m}=[x_{i,1},x_{i,2},\dots ,x_{i,m}]^T$. $y_i\in \mathbb {R}$ and ${\bar{x}}_{i,n}=[x_{i,1},x_{i,2},\dots ,x_{i,n}]^T$ are the system output and state vector. $f_{i,m}(\cdot )(m=1,\dots ,n)$ is the unknown nonlinear function. In this article, it is supposed that only $y_i$ is measurable and the leader’s signal $y_{jr}(j=N+1,\dots ,M+N)$ is a sufficiently smooth bounded function which first and two-order time derivatives are bounded.

Then, the system (3) can be rewritten as,

4 $$\begin{aligned} \left\{ \begin{array}{lll} \dot{x}_{i,1}=x_{i,2}+f_{i,1}({\hat{x}}_{i,1})+\Delta f_{i,1}, \\ \dot{x}_{i,m}=x_{i,m+1}+f_{i,m}(\hat{{\bar{x}}}_{i,m})+\Delta f_{i,m},\,\,\,2\le m\le n-1\\ \dot{x}_{i,n}=f_{i,n}(\hat{{\bar{x}}}_{i,n})+\Delta f_{i,n}+u_i, \\ y_i=x_{i,1} \end{array} \right. \end{aligned}$$

where $\Delta f_{i,1}=f_{i,1}(x_{i,1})-f_{i,1}({\hat{x}}_{i,1})$, $\Delta f_{i,m}=f_{i,m}(x_{i,m})-f_{i,m}({\hat{x}}_{i,m})$, $\Delta f_{i,n}=f_{i,n}(x_{i,n})-f_{i,n}({\hat{x}}_{i,m})$, ${\hat{x}}_{i,1}$ is the estimated values of $x_{i,1}$.

#### Control objective

This article aims to formula an observer-based fixed-time adaptive containment control law for the nonlinear MASs (3) to make the controlled systems are practically fixed-time stable, and all agents are able to converge to the convex hull formed by the leader agents in fixed-time, while maintaining a bounded containment error.

##### Lemma 2

Reference^45^ If $x_i\in \mathbb {R}$, $i=1,\dots ,\varrho$ and $\phi >1$, one has,

5 $$\begin{aligned} \Big (\sum _{i=1}^\varrho |x_i|\Big )^\phi\le & {} 2^{\phi -1}\sum _{i=1}^\varrho |x_i|^\phi , \end{aligned}$$

6 $$\begin{aligned} \Big (\sum _{i=1}^\varrho |x_i|\Big )^{1/\phi }\le & {} \sum _{i=1}^\varrho |x_i|^{1/\phi } \end{aligned}$$

##### Lemma 3

Reference^46^ For $x_1\in \mathbb {R}$, $x_2\in \mathbb {R}$, and a real number $c \ge 1$, one has,

7 $$\begin{aligned} |x_1+x_2|^c\le & {} 2^{c-1}|{\textrm{sig}}^c(x_1)+{\textrm{sig}}^c(x_2)|, \end{aligned}$$

8 $$\begin{aligned} |x_1-x_2|^c\le & {} 2^{c-1}|{\textrm{sig}}^c(x_1)-{\textrm{sig}}^c(x_2)| \end{aligned}$$

##### Lemma 4

Reference^45^ If $g_1>0$, $g_2>0$, and $\zeta >0$ , we have,

9 $$\begin{aligned} |x_1|^{g_1}|x_2|^{g_2}\le {g_1\zeta \over g_1+g_2}|x_1|^{g_1+g_2}+{g_2\zeta ^{-g_1/g_2}\over g_1+g_2}|x_2|^{g_1+g_2} \end{aligned}$$

##### Lemma 5

Reference^33^ The practical fixed-time stability of the nonlinear system $\dot{x}=\mathfrak {J}(x,t),\,\,x(0)=x_0,\,\,x\in \mathbb {R}^n$ can be obtained, if there is a Lyapunov function *V*, such that,

10 $$\begin{aligned} \dot{V}\le -(a V^{c_3}+bV^{c_4})^k+\Delta , \end{aligned}$$

where $c_3$, $c_4$, *a*, $b\in \mathbb {R}^+$, ${c_4}k>1$, $c_3k<1$, and $0<\Delta <\infty$. The residual set of the solution is described by,

11 $$\begin{aligned} \Big \{\lim _{t\rightarrow T}x|V\le \min \big \{a^{-{1\over c_3}}\left( {\Delta \over {1-\eta ^k}}\right) ^{1\over kc_3},b^{-{1\over c_4}}\left( {\Delta \over {1-\eta ^k}}\right) ^{1\over kc_4}\big \}\Big \} \end{aligned}$$

with $0<\eta <1$. The settling time can be bounded by,

12 $$\begin{aligned} T\le {1\over a^k\eta ^k(1-c_3k)}+{1\over b^k\eta ^k(c_4k-1)} \end{aligned}$$

##### Assumption 1

Each follower agent has at least one leader and the leader agents do not have neighbors.

##### Assumption 2

Based on the Assumption 1, the matrix $\mathfrak {T}_1$ is symmetric and positive definite, all entries of $-\mathfrak {T}_1^{-1}\mathfrak {T}_2$ is nonnegative, and each row of $-\mathfrak {T}_1\mathfrak {T}_2$ equal to 1.

##### Assumption 3

For $\forall$ $\mathfrak {X}_1$, $\mathfrak {X}_2\in \mathbb {R}^m$, the unknown function $h_{i,m}(\cdot )$ satisfies the following inequality,

13 $$\begin{aligned} |h_{i,m}(\mathfrak {X}_1)-h_{i,m}(\mathfrak {X}_2)|\le \iota _{i,m}\Vert \mathfrak {X}_1-\mathfrak {X}_2\Vert \end{aligned}$$

where $\iota _{i,m}$ is a constant.

##### Remark 1

In our study, the MASs under consideration are more practical and comprehensive, which involves unmeasurable states and unknown nonlinear function. Hence, compared with some recent works on containment control, this definition of the controlled systems is more general.

## State observer and containment control scheme design

### Design of state observer

To obtain the information of unmeasurable states, a state observer will be constructed.

Because the nonlinear term, $f_{i,m}(\cdot )\,(1\le m\le n)$ is unknown, the FLSs are utilized to identify them. According to Lemma 1, one has,

14 $$\begin{aligned} f_{i,m}(\hat{{\bar{x}}}_{i,m})=\theta _{i,m}^{*T}\varsigma _{i,m}(\hat{{\bar{x}}}_{i,m})+\varepsilon _{i,m} \end{aligned}$$

where $\hat{{\bar{x}}}_{i,m}=[\hat{x}_{i,1},\hat{x}_{i,2},\dots ,\hat{x}_{i,m}]$ denotes the estimation of ${{\bar{x}}}_{i,m}=[{x}_{i,1},{x}_{i,2},\dots , {x}_{i,m}]$. $\varepsilon _{i,m}$ satisfies $|\varepsilon _{i,m}|<\varepsilon _{i,m}^*$ with $\varepsilon _{i,m}^*>0$.

A state observer is developed as,

15 $$\begin{aligned} \left\{ \begin{array}{lll} {\dot{\hat{x}}}_{i,1}={\hat{x}}_{i,2}+k_{1}(z_{i,1}-{\hat{z}}_{i,1}), \\ {\dot{\hat{x}}}_{i,m}={{\hat{x}}}_{i,m+1}+k_{m}(z_{i,1}-{\hat{z}}_{i,1}),\,\,2\le m\le n-1\\ \dot{x}_{i,n}=k_{n}(z_{i,1}-{\hat{z}}_{i,1})+u_i \\ {\hat{y}}_i={\hat{x}}_{i,1} \end{array} \right. \end{aligned}$$

where $k_{i}>0 (i=1,\dots ,n)$ is designed parameter, $z_{i,1}$ is containment error, which is described by,

16 $$\begin{aligned} z_{i,1}=\sum _{j=1}^N\alpha _{ij}(y_i-y_j)+\sum _{j=N+1}^{N+M}\alpha _{ij}(y_i-y_{jr}(t)) \end{aligned}$$

The estimation of $z_{i,1}$ is defined as ${\hat{z}}_{i,1}$, which can described by,

17 $$\begin{aligned} {\hat{z}}_{i,1}=\sum _{j=1}^N\alpha _{ij}({\hat{y}}_i-{\hat{y}}_j)+\sum _{j=N+1}^{N+M}\alpha _{ij}({\hat{y}}_i-y_{jr}(t)) \end{aligned}$$

The observer error $e_i$ is constructed as,

18 $$\begin{aligned} e_i={\bar{x}}_{i,n}-\hat{{\bar{x}}}_{i,n}=[e_{i,1},e_{i,2},\dots ,e_{i,n}]^T \end{aligned}$$

With the help of (3) to (18), yields,

19 $$\begin{aligned} \dot{e}_i=Ke_{i,1}+Ae_i-K{\widetilde{z}}_{i,1}+\Delta f_{i}+\varepsilon _i+\sum _{m=1}^nB_{i,m}\theta _{i,m}^{*T}\varsigma _{i,m}(\hat{{\bar{x}}}_{i,m}) \end{aligned}$$

where

$$\begin{aligned} A=\left[ \begin{matrix} -k_{1} &{} 1 &{} 0 &{} \cdots &{} 0 \\ \vdots &{} \vdots &{} \vdots &{} \cdots &{} \vdots \\ -k_{n-1} &{} 0 &{} 0 &{} \cdots &{} 1 \\ -k_{n} &{} 0 &{} 0 &{} \cdots &{} 0 \end{matrix} \right] \in \mathbb {R}^{n\times n},\,\, B_{i,m}=[\underbrace{0\cdots 0\,\,\,1}_m\,\,\,0]^T\in \mathbb {R}^n \end{aligned}$$

$$K=[k_{1},k_{2},\dots ,k_{n}]^T$$, $$\Delta f_i=[\Delta f_{i,1},\Delta f_{i,2},\dots ,\Delta f_{i,n}]^T$$, $$\Delta f_{i,m}=f_{i,m}({\bar{x}}_{i,m})-f_{i,m}(\hat{{\bar{x}}}_{i,m})$$, $$\varepsilon _i=[\varepsilon _{i,1},\varepsilon _{i,2},\dots ,\varepsilon _{i,n}]^T$$.

Denote,

20 $$\begin{aligned} e=[e_1^T,\dots ,e_N^T]^T,\,\, \varepsilon =[\varepsilon _1^T,\dots ,\varepsilon _N^T]^T,\,\,e^1=[e_{1,1},e_{2,1},\dots ,e_{N,1}]^T,\,\,\Delta f=[\Delta f_1^T,\dots , \Delta f_N^T]^T. \end{aligned}$$

By invoking (19) and (20), one has,

21 $$\begin{aligned} \dot{e}=(I_N\otimes A)e+((I_N-\mathfrak {T}_1)\otimes K)e^1+\Delta f+\varepsilon +\Theta ^*\varsigma \end{aligned}$$

where $$\varsigma =[\varsigma _{1,1}^T,\dots ,\varsigma _{1,n}^T,\dots ,\varsigma _{N,1}^T,\dots ,\varsigma _{N,n}^T]^T$$, $$\Theta ^*={\textrm{diag}}[\theta _{1,1}^{*T},\dots ,\theta _{1,n}^{*T},\dots ,\theta _{N,1}^{*T},\dots ,\theta _{N,n}^{*T}]$$.

By designing vector *K* to confirm *A* is a strict Hurwitz matrix. Hence, for matrix $Q=Q^T>0$, one has,

22 $$\begin{aligned} \Upsilon A+A^T\Upsilon =-Q \end{aligned}$$

where $$\Upsilon =\Upsilon ^T>0$$.

The Lyapunov function is designed as,

23 $$\begin{aligned} V_o={1\over 2}e^T(I_N\otimes \Upsilon )e \end{aligned}$$

Then, the derivative of $\dot{V}_o$ is,

24 $$\begin{aligned} {\dot{V}}_o&={1\over 2}e^T(I_N\otimes (\Upsilon A+A^T\Upsilon ))e+e^T((I_N-\mathfrak {T}_1)\otimes \Upsilon K)e^1+e^T(I_N\otimes \Upsilon )(\Delta f+\varepsilon +\Theta ^{*T}\varsigma )\nonumber \\&\le -{1\over 2}e^T(I_N\otimes Q)e+\lambda _{\textrm{max}}(I_N-\mathfrak {T}_1)\lambda _{\textrm{max}}(\Upsilon K)\Vert e\Vert ^2+e^T(I_N\otimes \Upsilon )(\Delta f+\varepsilon +\Theta ^{*T}\varsigma ) \end{aligned}$$

where $\lambda _{\textrm{max}}(\cdot )$ denotes the maximum singular value of the corresponding matrix.

Under Assumption 3, we have,

25 $$\begin{aligned}&e^T(I_N\otimes \Upsilon )\Delta f\le {\textrm{max}}(\iota _{i,m})\lambda _{\textrm{max}}(\Upsilon )\Vert e\Vert ^2 \nonumber \\&e^T(I_N\otimes \Upsilon )\varepsilon \le {1\over 2}\lambda _{\textrm{max}}(\Upsilon )^2\Vert e\Vert ^2+{1\over 2}\Vert \varepsilon \Vert ^2 \nonumber \\&e^T(I_N\otimes \Upsilon )\Theta ^{*T}\varsigma \le {1\over 2}\lambda _{\textrm{max}}(\Upsilon )^2\Vert e\Vert ^2+{Nn\over 2}\Vert \Theta ^{*}\Vert ^2 \end{aligned}$$

According to (25), we have,

26 $$\begin{aligned} {\dot{V}}_o\le -\lambda _1\Vert e\Vert ^2+{Nn\over 2}\Vert \Theta ^{*}\Vert ^2+{1\over 2}\Vert \varepsilon \Vert ^2 \end{aligned}$$

where $\lambda _1=\lambda _{\textrm{min}}(Q)/2-\lambda _{\textrm{max}}(I_N-\mathfrak {T}_1)\lambda _{\textrm{max}}(\Upsilon K)-{\textrm{max}}(\iota _{i,m})\lambda _{\textrm{max}}(\Upsilon )-\lambda _{\textrm{max}}(\Upsilon )^2>0$, $\lambda _{\textrm{min}}(\cdot )$ refers to the minimum singular value of the corresponding matrix.

#### Remark 2

Note that this paper constructs a state observer to obtain each follower’s state information. Based on (23) and (26), we know that the estimate errors are bounded, which means there exists ${\bar{e}}_{i,m}>0$ satisfying $|e_{i,m}|<{\bar{e}}_{i,m}$. Thus, we can see that the error *e* satisfies $\Vert e\Vert <{\bar{e}}$ with ${\bar{e}}=\Vert [{\bar{e}}_{1},\dots , {\bar{e}}_{m}]\Vert$, ${\bar{e}}_{m}=\Vert [{\bar{e}}_{1,m},\dots ,{\bar{e}}_{i,m}]\Vert$. Furthermore, it is apparent that the estimation errors exhibit practical fixed-time stability, which will be proved by the stability analysis later.

### Design of containment controller

In this section, an adaptive backstepping-based fixed-time containment control protocol will be developed, which derive from the adding one power integrator approach.

Defined the coordinate transformation as follows,

27 $$\begin{aligned} z_{i,m}={\textrm{sig}}^{1/\gamma _m}({\hat{x}}_{i,m})-{\textrm{sig}}^{1/\gamma _m}(v_{i,m}),\,\,\,m=2,\dots ,n \end{aligned}$$

where $1>\gamma _m=\gamma _{m-1}+\tau >0$, $\gamma _1=1$, $0>\tau >-1$, $\mathrm{{sig}}^{1/\gamma _m}({\hat{x}}_{i,m})=\mathrm{{sign}}({\hat{x}}_{i,m})|{\hat{x}}_{i,m}|^{1/\gamma _m}$, $v_{i,m}$ is virtual control law, $\mathrm{{sig}}^{1/\gamma _m}(v_{i,m})=\mathrm{{sign}}(v_{i,m})|{\hat{v}}_{i,m}|^{1/\gamma _m}$.

#### Remark 3

In reference^47^, a fuzzy finite-time adaptive control protocol based on the adding one power integrator approach was derived for nonstrict-feedback systems with unknown nonlinear functions. In reference^48^, via employing homogeneous system theory, adding one power integrator approach, and nested saturation control method, a fixed-time control algorithm was developed for switched nonstrict-feedback systems with unknown functions and actuator failure. Note that the formulated controllers mentioned above by merging the adding one power integrator approach where the coordinate transformations are defined as $z_{i,m}={{\hat{x}}_{i,m}}^{1/\gamma _m}-{v_{i,m}}^{1/\gamma _m}$. For these coordinate transformation, the power should be a ratio of two odd numbers or an odd number. In this article by using the sign function, a new coordinate transformation is defined as $z_{i,m}={\textrm{sig}}^{1/\gamma _m}({\hat{x}}_{i,m})-{\textrm{sig}}^{1/\gamma _m}(v_{i,m})$ in which the power does not need to satisfy this limitation.

*Step 1*: From (16) and $x_{i,2}=e_{i,2}+{\hat{x}}_{i,2}$, we have

28 $$\begin{aligned} \dot{z}_{i,1}=\varrho _i(x_{i,2}+\Delta f_{i,1}+f_{i,1}({\hat{x}}_{i,1})+f_{ij,1}({\hat{x}}_{ij,1}))-\sum _{j=N+1}^{N+M}\alpha _{ij}{\dot{y}}_{jr}- \sum _{j=1}^N\alpha _{ij}(e_{j,2}+{\hat{x}}_{j,2}+\Delta f_{j,1}) \end{aligned}$$

where $f_{ij,1}({\hat{x}}_{ij,1})=\sum _{j=1}^Nf_{j,1}({\hat{x}}_{j,1})/\varrho _i$ and $\varrho _i=\sum _{j=1}^N\alpha _{ij}+\sum _{j=N+1}^{N+M}\alpha _{ij}$ . By Lemma 1, the FLSs are developed to identify the $f_{ij,1}({\hat{x}}_{ij,1})$ and $f_{i,1}({\hat{x}}_{i,1})$, which can be described as

29 $$\begin{aligned} \left\{ \begin{array}{lll} f_{i,1}({\hat{x}}_{i,1})=\theta _{i,1}^{*T}\varsigma _{i,1}(\hat{x}_{i,1})+\varepsilon _{i,1}\\ f_{ij,1}({\hat{x}}_{ij,1})=\theta _{ij,1}^{*T}\varsigma _{ij,1}(\hat{ x}_{ij,1})+\varepsilon _{ij,1} \end{array} \right. , \end{aligned}$$

Design the following Lyapunov function

30 $$\begin{aligned} V_{i,1}={1\over 2}z_{i,1}^2+{1\over 2}{{{\widetilde{\Im }}}^2}_i \end{aligned}$$

By invoking (28) and (29), it has

31 $$\begin{aligned} \dot{V}_{i,1}&=z_{i,1}\big (\varrho _i(e_{i,2}+{\hat{x}}_{i,2}-v_{i,2}+v_{i,2}+\theta _{i,1}^{*T}\varsigma _{i,1}(\hat{x}_{i,m})+\varepsilon _{i,1}+\theta _{ij,1}^{*T}\varsigma _{ij,1}(\hat{x}_{ij,1})+\varepsilon _{ij,1}+\Delta f_{i,1})\nonumber \\&\quad -\sum _{j=1+N}^{M+N}\alpha _{ij}{\dot{y}}_{jr}- \sum _{j=1}^N\alpha _{ij}(e_{j,2}+{\hat{x}}_{j,2}+\Delta f_{j,1})\big )+{{\widetilde{\Im }}}_i\dot{{\widetilde{\Im }}}_i \end{aligned}$$

According to Lemma 4, it has

32 $$\begin{aligned} z_{i,1}\varrho _ie_{i,2}&\le {\mu _1\over 2}\varrho _i^2z_{i,1}^2+{1\over 2\mu _1}\Vert e\Vert ^2,\,\,\,-z_{i,1}\sum _{j=1}^N\alpha _{ij}e_{j,2}\le {\mu _2\over 2}z_{i,1}^2+{1\over 2\mu _2}\Vert e\Vert ^2\nonumber \\ z_{i,1}\varrho _i\Delta f_{i,1}&\le \iota _{i,m}|z_{i,1}||e_{i,1}|\le {\mu _3\over 2}\iota _{i,1}^2z_{i,1}^2+{1\over 2\mu _3}\Vert e\Vert ^2,\,\,\,-z_{i,1}\sum _{j=1}^N\alpha _{ij}\Delta f_{j,1}\le {\mu _4\over 2}\iota _{j,1}^2z_{i,1}^2+{1\over 2\mu _4}\Vert e\Vert ^2 \end{aligned}$$

where $\mu _1$, $\mu _2$, $\mu _3$, and $\mu _4$ are positive constants.

Based on (29), one has

33 $$\begin{aligned} \theta _{i,1}^{*T}\varsigma _{i,1}(\hat{x}_{i,1})+\varepsilon _{i,1}\le {\bar{\theta }}_{i,1}^{*T}{\bar{\varsigma }}_{i,1}(\hat{x}_{i,1}) \end{aligned}$$

where $|\varepsilon _{i,1}|\le \varepsilon _{i,1}^*$ with $\varepsilon _{i,1}^*>0$. ${\bar{\varsigma }}_{i,1}^T(\hat{x}_{i,1})=[\varsigma _{i,1}^T(\hat{ x}_{i,1}),1]$, and ${\bar{\theta }}_{i,1}^{*T}=[\theta _{i,1}^{*T}, \varepsilon _{i,1}^*]$. Based on Lemma 4 and noting that $0<\varsigma _{i,1}^T(\cdot )\varsigma _{i,1}(\cdot )\le 1$, one has

34 $$\begin{aligned} z_{i,1}\varrho _i(\theta _{i,1}^{*T}\varsigma _{i,1}+\varepsilon _{i,1})\le&\varrho _i|z_{i,1}|\zeta _{i,1}^{1\over 2} \le \varrho _i|z_{i,1}|\Im _i^{*{1\over 2}} \le {\bar{\gamma }}_1z_{i,1}^2\Im _i^{*}+\Gamma _{i,1} \end{aligned}$$

where $$\zeta _{i,1}=(\sqrt{2}\Vert {\bar{\theta }}_{i,1}^{*T}\Vert )^2$$, $${\bar{\gamma }}_1=\varrho _i{o_1\over 2}$$, $$\Im _i^{*}={\mathrm{\max }}\{\zeta _{i,1},\dots \zeta _{i,n},\zeta _{ij,1},\zeta _{i,1s}\}$$, $$\Gamma _{i,1}={\varrho _i\over 2o_1}$$ with $$o_1>0$$.

Similar to (34), we have

35 $$\begin{aligned} z_{i,1}\varrho _i(\theta _{ij,1}^{*T}\varsigma _{ij,1}+\varepsilon _{ij,1})\le&\varrho _i|z_{i,1}|\zeta _{ij,1}^{1\over 2} \le \varrho _i|z_{i,1}|\Im _i^{*{1\over 2}} \le {\bar{\gamma }}_{1}z_{i,1}^2\Im _i^{*}+\Gamma _{i,1} \end{aligned}$$

Applying Lemma 2 and 3 yields

36 $$\begin{aligned} z_{i,1}({\hat{x}}_{i,2}-v_{i,2})\le |{\hat{x}}_{2}-v_{i,2}||z_{i,1}|\le 2|z_{i,1}||z_{i,2}|^{\gamma _2} \end{aligned}$$

Let $F_{i,1}(S_{i,1})=2|z_{i,2}|^{\gamma _2}+|\sum _{j=1+N}^{M+N}\alpha _{ij}{\dot{y}}_{jr}/\varrho _i|+|\sum _{j=1}^N\alpha _{ij}{\hat{x}}_{j,2}/\varrho _i|$ and $S_{i,1}=[z_{i,2},{\dot{y}}_{j0},{\hat{x}}_{j,2}]^T$. Subsequently, utilizing the FLSs to approximate the nonlinear function $F_{i,1}$, we have

37 $$\begin{aligned} F_{i,1}(S_{i,1})=\theta _{i,1s}^{*T}\varsigma _{i,1s}+\varepsilon _{i,1s} \end{aligned}$$

Similar to (34) and (35), we have

38 $$\begin{aligned} |z_{i,1}|\varrho _i(\theta _{i,1s}^{*T}\varsigma _{i,1s}(S_{i,1})+\varepsilon _{i,1s})\le&\varrho _i|z_{i,1}|\zeta _{i,1s}^{1\over 2} \le \varrho _i|z_{i,1}|\Im _i^{*{1\over 2}} \le {\bar{\gamma }}_1z_{i,1}^2\Im _i^{*}+\Gamma _{i,1} \end{aligned}$$

From inequalities (32) to (38), yields

39 $$\begin{aligned} \dot{V}_{i,1}\le {\bar{\mu }}_2\Vert e\Vert ^2+z_{i,1}\varrho _iv_{i,2}+{\bar{\mu }}_{i,1}z_{i,1}^2 +\chi z_{i,1}^2\Im _i^{*}+3\Gamma _{i,1}+{{\widetilde{\Im }}}_i\dot{{\widetilde{\Im }}}_i \end{aligned}$$

where $${\bar{\mu }}_{i,1}={\mu _1\over 2}\varrho _i^2+{\mu _2\over 2}+{\mu _3\over 2}\iota _{i,1}^2+{\mu _4\over 2}\iota _{j,1}^2$$, $${\bar{\mu }}_2={1\over 2\mu _1}+{1\over 2 \mu _2}+{1\over 2\mu _3}+{1\over 2\mu _4}$$, $$\chi =3{\bar{\gamma }}_1$$.

The virtual controller $v_{i,2}$ is derived as,

40 $$\begin{aligned} v_{i,2}={1\over \varrho _i}\big (-n_{1,1}{\textrm{sig}}^{2+\tau \over 2-\tau }(z_{i,1})-n_{1,2}{\textrm{sig}}^{6+\tau \over 2-\tau }(z_{i,1})-{\bar{\mu }}_{i,1}z_{i,1}-\chi z_{i,1}{\hat{\Im }}_i\big ) \end{aligned}$$

where ${{\hat{\Im }}}_i$ is the estimation of the $\Im ^{*}_i$. By invoking (40)-(75), one can have,

41 $$\begin{aligned} \dot{V}_{i,1}&\le -n_{1,1}|z_{i,1}|^{4\over 2-\tau }+{\bar{\mu }}_2\Vert e\Vert ^2-n_{1,2}|z_{i,1}|^{8\over 2-\tau }-\chi z_{i,1}^2{\hat{\Im }}_i+\chi z_{i,1}^2\Im ^{*}_i-{{\widetilde{\Im }}}_i\dot{{\hat{\Im }}}_i+3\Gamma _{i,1}\nonumber \\&\le {\bar{\mu }}_2\Vert e\Vert ^2-n_{1,2}|z_{i,1}|^{8\over 2-\tau }-n_{1,1}|z_{i,1}|^{4\over 2-\tau }-{\widetilde{\Im }}_i\dot{{\hat{\Im }}}_i+{\widetilde{\Im }}_i\chi z_{i,1}^2+3\Gamma _{i,1} \end{aligned}$$

*Step 2*: Consider the Lyapunov function as:

42 $$\begin{aligned} V_{i,2}=V_{i,1}+\varpi _{i,2} \end{aligned}$$

where

43 $$\begin{aligned} \varpi _{i,2}=\int _{v_{i,2}}^{{\hat{x}}_{i,2}}{\textrm{sig}}^{2-\gamma _{3}}\big ({\textrm{sig}}^{1/\gamma _2}(s)-{\textrm{sig}}^{1/ \gamma _2}({v_{i,2}})\big )ds \end{aligned}$$

According to the integra derivation rule, the following equations holds:

44 $$\begin{aligned} {\partial \varpi _{i,2}\over \partial {\hat{x}}_{i,2}}&={\textrm{sig}}^{2-\gamma _{3}}(z_{i,2})\nonumber \\ {\partial \varpi _{i,2}\over \partial \Lambda }&=-(2-\gamma _{3})\int _{v_{i,2}}^{{\hat{x}}_{i,2}}{\textrm{sig}}^{1-\gamma _{3}}\big ({\textrm{sig}}^{1/\gamma _2}(s)-{\textrm{sig}}^{1/ \gamma _2}({v_{i,2}})\big )ds{\partial {\textrm{sig}}^{1/\gamma _2}(v_{i,2})\over \partial \Lambda } \end{aligned}$$

where $``\Lambda ''$ is ${\hat{\Im }}_i$ and ${\hat{x}}_{i,1}$.

Furthermore, we have,

45 $$\begin{aligned} |{\partial \varpi _{i,2}\over \partial \Lambda }|&\le (2-\gamma _{3})\left| \int _{v_{i,2}}^{{\hat{x}}_{i,2}}{\textrm{sig}}^{1-\gamma _{3}}\big ({\textrm{sig}}^{1/\gamma _2}(s)-{\textrm{sig}}^{1/ \gamma _2}({v_{i,2}})\big )ds\right| \left| {\partial {\textrm{sig}}^{1/\gamma _2}(v_{i,2})\over \partial \Lambda }\right| \nonumber \\&\le 2(2-\gamma _{3})|z_{i,2}|^{1-\tau }\left| {\partial {\textrm{sig}}^{1/\gamma _2}(v_{i,2})\over \partial \Lambda }\right| \end{aligned}$$

Then, the derivative of $V_{i,2}$ is,

46 $$\begin{aligned} \dot{V}_{i,2}&=\dot{V}_{i,1}+z_{i,2}^{2-\gamma _{3}}{\dot{\hat{x}}}_{i,2}+\sum _{k=1}^{2}{\partial \varpi _{i,2}\over \partial {{\hat{x}}}_{i,k}}{\dot{\hat{x}}}_{i,k}+{\partial \varpi _{i,2}\over \partial {\hat{\varphi }}}\dot{{\hat{\varphi }}}\nonumber \\&=\dot{V}_{i,1}+{\textrm{sig}}^{2-\gamma _{3}}(z_{i,2})\big ({{\hat{x}}}_{i,3}+v_{i,3}-v_{i,3}+k_2(z_{i,1}-{\hat{z}}_{i,1})\big )+\sum _{k=1}^{2}{\partial \varpi _{i,2}\over \partial {{\hat{x}}}_{i,k}}{\dot{\hat{x}}}_{i,k}+{\partial \varpi _{i,2}\over \partial {\hat{\Im }}_i}\dot{{\hat{\Im }}}_i\nonumber \\&\le \dot{V}_{i,1}+{\textrm{sig}}^{2-\gamma _{3}}(z_{i,2})\big ({{\hat{x}}}_{i,3}-v_{i,3}+v_{i,3}+k_2(z_{i,1}-{\hat{z}}_{i,1})\big )+2(2-\gamma _{3})\left| {\partial {\textrm{sig}}^{1/\gamma _2}(v_{i,2})\over \partial \Lambda }\right| |z_{i,2}|^{1-\tau } \end{aligned}$$

Applying Lemma 2 and 3 yields,

47 $$\begin{aligned} |{{\hat{x}}}_{i,3}-v_{i,3}|\le 2^{1-\gamma _{3}}|{\textrm{sig}}^{1/\gamma _{3}}({{\hat{x}}}_{i,3})-{\textrm{sig}}^{1/\gamma _{3}}(v_{i,3})|\le 2|z_{i,3}|^{\gamma _{3}} \end{aligned}$$

Consequently, it follows from Lemma 4 that,

48 $$\begin{aligned} |({{\hat{x}}}_{i,3}-v_{i,3}){\textrm{sig}}^{2-\gamma _{3}}(z_{i,2})|&\le 2|z_{i,2}|^{2-\gamma _{3}}|z_{i,m+1}|^{\gamma _{3}}\le \mathfrak {C}_{i,3}|z_{i,3}|^2+z_{i,2}^2 \end{aligned}$$

where $$\mathfrak {C}_{i,3}=\gamma _{3}(2-\gamma _{3})^{(2-\gamma _{3})/\gamma _{3}}>0$$.

49 $$\begin{aligned} \dot{V}_{i,2} \le \dot{V}_{i,1}+{\textrm{sig}}^{2-\gamma _{3}}(z_{i,2})(v_{i,3}+k_m(z_{i,1}-{\hat{z}}_{i,1})\big )+\mathfrak {C}_{i,3}|z_{i,3}|^2+z_{i,2}^2+2(2-\gamma _{3})|z_{i,2}|^{1-\tau }\left| {{\textrm{sig}}^{1/\gamma _2}(v_{i,2})\over \partial \Lambda }\right| \end{aligned}$$

Let $F_{i,2}(S_{i,2})=2(2-\gamma _{3})|{{\textrm{sig}}^{1/\gamma _2}(v_{i,2})\over \partial \Lambda }|$ where $S_{i,2}=[{\hat{x}}_{i,2},{\hat{\Im }}_i]^T$. Subsequently, utilizing the FLSs to approximate the nonlinear term $F_{i,2}$, we have,

50 $$\begin{aligned} F_{i,2}(S_{i,2})=\theta _{i,2s}^{*T}\varsigma _{i,2s}+\varepsilon _{i,2s}\le {\bar{\theta }}_{i,2s}^{*T}{\bar{\varsigma }}_{i,2s}(S_{i,2}) \end{aligned}$$

where $|\varepsilon _{i,2s}|\le \varepsilon _{i,2s}^*$ with $\varepsilon _{i,2s}^*>0$ . ${\bar{\theta }}_{i,2s}^{*T}=[\theta _{i,2s}^{*T}, \varepsilon _{i,2s}^*]$, and ${\bar{\varsigma }}_{i,2s}^T(S_{i,2})=[\varsigma _{i,2s}^T(S_{i,2}),1]$. According to Lemma 4 and noting that $0<\varsigma _{i,2s}^T(\cdot )\varsigma _{i,2s}(\cdot )\le 1$, one has

51 $$\begin{aligned} |z_{i,2}|^{1-\tau }(\theta _{i,2s}^{*T}\varsigma _{i,2s}+\varepsilon _{i,2s})\le&\zeta _{i,2}^{1-\tau \over 2} |z_{i,2}|^{1-\tau } \le \Im ^{*{1-\tau \over 2}}_i|z_{i,2}|^{1-\tau } \le \chi z_{i,2}^2\Im ^{*}_i+{\bar{\Gamma }} \end{aligned}$$

where $${\bar{\Gamma }}={1+\tau \over 2}({2\over 1-\tau } \chi )^{1-\chi \over 1+\chi }$$, $$\zeta _{i,2}=(\sqrt{2}\Vert {\bar{\theta }}_{i,2s}^{*T}\Vert )^{2/(1-\tau )}$$.

52 $$\begin{aligned} \dot{V}_{i,2} \le \dot{V}_{i,1}+{\textrm{sig}}^{2-\gamma _{3}}(v_{i,3}+k_2(z_{i,1}-{\hat{z}}_{i,1})\big )+\chi z_{i,2}^2\Im ^{*}_i+\mathfrak {C}_{i,3}|z_{i,3}|^2+{\bar{\Gamma }}+z_{i,2}^2 \end{aligned}$$

The virtual controller $v_{i,3}$ is constructed as,

53 $$\begin{aligned} v_{i,3}=-n_{2,1}{\textrm{sig}}^{2+\gamma _{3}}(z_{i,2})-n_{2,2}{\textrm{sig}}^{\gamma _{3}}(z_{i,2})-{\textrm{sig}}^{\gamma _{3}}(z_{i,2})\chi {\hat{\Im }}_i-k_2(z_{i,1}-{\hat{z}}_{i,1}) \end{aligned}$$

Then one has,

54 $$\begin{aligned} \dot{V}_{i,2}&\le \dot{V}_{i,1}-(n_{2,2}-1)z_{i,2}^2-n_{2,1}z_{i,2}^4-z_{i,2}^2\chi {\hat{\Im }}_i +\chi z_{i,2}^2\Im ^{*}_i+{\bar{\Gamma }}+\mathfrak {C}_{i,3}|z_{i,3}|^2\nonumber \\&\le -n_{1,1}|z_{i,1}|^{4\over 2-\tau }-n_{1,2}|z_{i,1}|^{8\over 2-\tau }+{\widetilde{\Im }}_i(\chi z_{i,1}^2+\chi z_{i,2}^2-\dot{{\hat{\Im }}}_i)-(n_{2,2}-1)z_{i,m}^2\nonumber \\&\quad -n_{2,1}z_{i,2}^4+(n-2){\bar{\Gamma }}+3\Gamma _{i,1}+\mathfrak {C}_{i,3}|z_{i,3}|^2+{\bar{\mu }}_2\Vert e\Vert ^2 \end{aligned}$$

where $$n_{2,2}>1$$.

*Step* *m*($3\le m\le n-1$): Define the following Lyapunov function:

55 $$\begin{aligned} V_{i,m}=V_{i,m-1}+\varpi _{i,m} \end{aligned}$$

where

56 $$\begin{aligned} \varpi _{i,m}=\int _{v_{i,m}}^{{\hat{x}}_{i,m}}{\textrm{sig}}^{2-\gamma _{m+1}}\big ({\textrm{sig}}^{1/\gamma _m}(s)-{\textrm{sig}}^{1/ \gamma _m}({v_{i,m}})\big )ds \end{aligned}$$

According to the integra derivation rule, the following equations holds:

57 $$\begin{aligned} {\partial \varpi _{i,m}\over \partial {\hat{x}}_{i,m}}&={\textrm{sig}}^{2-\gamma _{m+1}}(z_{i,m})\nonumber \\ {\partial \varpi _{i,m}\over \partial \Lambda }&=-(2-\gamma _{m+1})\int _{v_{i,m}}^{{\hat{x}}_{i,m}}{\textrm{sig}}^{1-\gamma _{m+1}}\big ({\textrm{sig}}^{1/\gamma _m}(s)-{\textrm{sig}}^{1/ \gamma _m}({v_{i,m}})\big )ds{\partial {\textrm{sig}}^{1/\gamma _m}(v_{i,m})\over \partial \Lambda } \end{aligned}$$

where $``\Lambda ''$ is ${\hat{\Im }}_i$ and ${\hat{x}}_{i,k}$, $k=1,\dots ,m-1$.

Furthermore, we have,

58 $$\begin{aligned} |{\partial \varpi _{i,m}\over \partial \Lambda }|&\le (2-\gamma _{m+1})\left| \int _{v_{i,m}}^{{\hat{x}}_{i,m}}{\textrm{sig}}^{1-\gamma _{m+1}}\big ({\textrm{sig}}^{1/\gamma _m}(s)-{\textrm{sig}}^{1/ \gamma _m}({v_{i,m}})\big )ds\right| \left| {\partial {\textrm{sig}}^{1/\gamma _m}(v_{i,m})\over \partial \Lambda }\right| \nonumber \\&\le 2(2-\gamma _{m+1})|z_{i,m}|^{1-\tau }\left| {\partial {\textrm{sig}}^{1/\gamma _m}(v_{i,m})\over \partial \Lambda }\right| \end{aligned}$$

Then, differentiating $V_{i,m}$ leads to,

59 $$\begin{aligned} \dot{V}_{i,m}&=\dot{V}_{i,m-1}+{\partial \varpi _{i,m}\over \partial {\hat{\varphi }}}\dot{{\hat{\varphi }}}+\sum _{k=1}^{n-1}{\partial \varpi _{i,m}\over \partial {{\hat{x}}}_{i,k}}{\dot{\hat{x}}}_{i,k}+z_{i,m}^{2-\gamma _{m+1}}{\dot{\hat{x}}}_{i,m}\nonumber \\&=\dot{V}_{i,m-1}+{\textrm{sig}}^{2-\gamma _{m+1}}(z_{i,m})\big (k_m(z_{i,1}-{\hat{z}}_{i,1})+{{\hat{x}}}_{i,m+1}-v_{i,m+1}+v_{i,m+1}\big )+{\partial \varpi _{i,m}\over \partial {\hat{\Im }}_i}\dot{{\hat{\Im }}}_i+\sum _{k=1}^{n-1}{\partial \varpi _{i,m}\over \partial {{\hat{x}}}_{i,k}}{\dot{\hat{x}}}_{i,k}\nonumber \\&\le \dot{V}_{i,m-1}+{\textrm{sig}}^{2-\gamma _{m+1}}(z_{i,m})\big ({{\hat{x}}}_{i,m+1}-v_{i,m+1}+v_{i,m+1}+k_m(z_{i,1}-{\hat{z}}_{i,1})\big )\nonumber \\&\quad +2(2-\gamma _{m+1})\left| {\partial {\textrm{sig}}^{1/\gamma _m}(v_{i,m})\over \partial \Lambda }\right| |z_{i,m}|^{1-\tau } \end{aligned}$$

Applying Lemma 2 and 3 yields,

60 $$\begin{aligned} |{{\hat{x}}}_{i,m+1}-v_{i,m+1}|\le 2^{1-\gamma _{m+1}}|{\textrm{sig}}^{1/\gamma _{m+1}}({{\hat{x}}}_{i,m+1})-{\textrm{sig}}^{1/\gamma _{m+1}}(v_{i,m+1})|\le 2|z_{i,m+1}|^{\gamma _{m+1}} \end{aligned}$$

Consequently, it follows from Lemma 4 that,

61 $$\begin{aligned} |({{\hat{x}}}_{i,m+1}-v_{i,m+1}){\textrm{sig}}^{2-\gamma _{m+1}}(z_{i,m})|&\le 2|z_{i,m+1}|^{\gamma _{m+1}}|z_{i,m}|^{2-\gamma _{m+1}}\nonumber \\&\le \mathfrak {C}_{i,m+1}z_{i,m+1}^2+z_{i,m}^2 \end{aligned}$$

where $$\mathfrak {C}_{i,m+1}=(2-\gamma _{m+1})^{2-\gamma _{m+1}\over \gamma _{m+1}}\cdot \gamma _{m+1}>0$$.

62 $$\begin{aligned} \dot{V}_{i,m}&\le \dot{V}_{i,m-1}+{\textrm{sig}}^{2-\gamma _{m+1}}(z_{i,m})(k_m(z_{i,1}-{\hat{z}}_{i,1})+v_{i,m+1}\big )+\mathfrak {C}_{i,m+1}z_{i,m+1}^2+z_{i,m}^2\nonumber \\&\quad +2(2-\gamma _{m+1})\left| {{\textrm{sig}}^{1/\gamma _m}(v_{i,m})\over \partial \Lambda }\right| |z_{i,m}|^{1-\tau } \end{aligned}$$

Let $F_{i,m}(S_{i,m})=-\mathfrak {C}_{i,m}|z_{i,m}|^{1+\tau }+2(2-\gamma _{m+1})|{{\textrm{sig}}^{1/\gamma _m}(v_{i,m})\over \partial \Lambda }|$ where $S_{i,m}=[{\hat{x}}_{i,m},{\hat{\Im }}_i]^T$. Subsequently, utilizing the FLSs to identify the nonlinear term $F_{i,m}$, we have

63 $$\begin{aligned} F_{i,m}=\theta _{i,ms}^{*T}\varsigma _{i,ms}+\varepsilon _{i,ms}\le {\bar{\theta }}_{i,ms}^{*T}{\bar{\varsigma }}_{i,ms} \end{aligned}$$

where $|\varepsilon _{i,ms}|\le \varepsilon _{i,ms}^*$ with $\varepsilon _{i,ms}^*>0$. ${\bar{\theta }}_{i,ms}^{*T}=[\theta _{i,ms}^{*T}, \varepsilon _{i,ms}^*]$, and ${\bar{\varsigma }}_{i,ms}^T(S_{i,m})=[\varsigma _{i,ms}^T(S_{i,m}),1]$. According to Lemma 4 and noting that $0<\varsigma _{i,ms}^T(\cdot )\varsigma _{i,ms}(\cdot )\le 1$, one has

64 $$\begin{aligned} |z_{i,m}|^{1-\tau }(\theta _{i,ms}^{*T}\varsigma _{i,ms}+\varepsilon _{i,ms})&\le |z_{i,m}|^{1-\tau }\zeta _{i,m}^{1-\tau \over 2}\nonumber \\&\le |z_{i,m}|^{1-\tau }\Im ^{*{1-\tau \over 2}}_i\nonumber \\&\le \chi z_{i,m}^2\Im ^{*}_i+{\bar{\Gamma }} \end{aligned}$$

where $$\zeta _{i,m}=(\sqrt{2}\Vert {\bar{\theta }}_{i,ms}^{*T}\Vert )^{2/(1-\tau )}$$, $${\bar{\Gamma }}={1+\tau \over 2}({2\over 1-\tau } \chi )^{1-\chi \over 1+\chi }$$.

65 $$\begin{aligned} \dot{V}_{i,m} \le \dot{V}_{i,m-1}+{\textrm{sig}}^{2-\gamma _{m+1}}(v_{i,m+1}+k_m(z_{i,1}-{\hat{z}}_{i,1})\big )+\chi z_{i,m}^2\Im ^{*}_i+{\bar{\Gamma }}+\mathfrak {C}_{i,m+1}z_{i,m+1}^2+z_{i,m}^2 \end{aligned}$$

The virtual control protocol $v_{i,m+1}$ is developed as,

66 $$\begin{aligned} v_{i,m+1}=-n_{m,1}{\textrm{sig}}^{2+\gamma _{m+1}}(z_{i,m})-n_{m,2}{\textrm{sig}}^{\gamma _{m+1}}(z_{i,m})-{\textrm{sig}}^{\gamma _{m+1}}(z_{i,m})\chi {\hat{\Im }}_i-k_m(z_{i,1}-{\hat{z}}_{i,1}) \end{aligned}$$

Then we have

67 $$\begin{aligned} \dot{V}_{i,m}&\le \dot{V}_{i,m-1}-(n_{m,2}-1)z_{i,m}^2-n_{m,1}z_{i,m}^4-z_{i,m}^2\chi {\hat{\Im }}_i +\chi z_{i,m}^2\Im ^{*}_i+{\bar{\Gamma }}+\mathfrak {C}_{i,m+1}z_{i,m+1}^2\nonumber \\&\le -n_{1,1}|z_{i,1}|^{4\over 2-\tau }-n_{1,2}|z_{i,1}|^{8\over 2-\tau }-\sum _{m=2}^{n-1}(n_{m,2}-1)z_{i,m}^2+{\widetilde{\Im }}_i\left( \sum _{m=1}^{n-1}\chi z_{i,m}^2-\dot{{\hat{\Im }}}_i\right) \nonumber \\&\quad -\sum _{m=2}^{n-1}n_{m,1}z_{i,m}^4+(n-2){\bar{\Gamma }}+3\Gamma _{i,1}+{\bar{\mu }}_2\Vert e\Vert ^2+\mathfrak {C}_{i,m+1}z_{i,m+1}^2 \end{aligned}$$

where $$n_{m,2}>1$$.

*Step* *n*: Consider the following Lyapunov function:

68 $$\begin{aligned} V_{i,n}=V_{i,n-1}+\varpi _{i,n} \end{aligned}$$

where

69 $$\begin{aligned} \varpi _{i,n}=\int _{v_{i,n}}^{{\hat{x}}_{i,n}}{\textrm{sig}}^{2-\gamma _{n+1}}\big ({\textrm{sig}}^{1/\gamma _n}(s)-{\textrm{sig}}^{1/ \gamma _n}({v_{i,n}})\big )ds \end{aligned}$$

Then, the differentiating $\dot{V}_{i,n}$ leads to,

70 $$\begin{aligned} \dot{V}_{i,n}&=\dot{V}_{i,n-1}+{\textrm{sig}}^{2-\gamma _{n+1}}(z_{i,n}){\dot{\hat{x}}}_{i,n}+{\partial \varpi _{i,n}\over \partial {{\hat{\Im }}}_i}\dot{{\hat{\Im }}}_i+\sum _{k=1}^{n}{\partial \varpi _{i,n}\over \partial {{\hat{x}}}_{i,k}}{\dot{\hat{x}}}_{i,k}\nonumber \\&=\dot{V}_{i,n-1}+{\textrm{sig}}^{2-\gamma _{n+1}}(z_{i,n})\big (u_i+k_n(z_{i,1}-{\hat{z}}_{i,1})\big )+\sum _{k=1}^{n}{\partial \varpi _{i,n}\over \partial {{\hat{x}}}_{i,k}}{\dot{\hat{x}}}_{i,k}+{\partial \varpi _{i,n}\over \partial {\hat{\Im }}}_i\dot{{\hat{\Im }}}_i\nonumber \\&\le -n_{1,1}|z_{i,1}|^{4\over 2-\tau }-n_{1,2}|z_{i,1}|^{8\over 2-\tau }+{\widetilde{\Im }}_i\left( \sum _{m=1}^{n-1}\chi z_{i,m}^2-\dot{{\hat{\Im }}}_i\right) -\sum _{m=2}^{n-1}(n_{m,2}-1)z_{i,m}^2\nonumber \\&\quad -\sum _{m=2}^{n-1}n_{m,1}z_{i,m}^4+{\textrm{sig}}^{2-\gamma _{n+1}}(z_{i,n})\big (u_i+k_n(z_{i,1}-{\hat{z}}_{i,1})\big )+|z_{i,n}|^{1-\tau }F_{i,n}(S_{i,n})\nonumber \\&\quad +({n-2}){\bar{\Gamma }}+3\Gamma _{i,1}+{\bar{\mu }}_2\Vert e\Vert ^2 \end{aligned}$$

where $F_{i,n}(S_{i,n})=\mathfrak {C}_{i,n}|z_{i,n}|^{1+\tau }+2(2-\gamma _{n+1})|{\partial {\textrm{sig}}^{1/\gamma _n}(v_{i,n})\over \partial \Lambda }|$ with $S_{i,n}=[{\hat{x}}_{i,n},{\hat{\Im }}_i]^T$. Then, according to Lemma 1, $F_{i,n}(S_{i,n})$ can be described by,

71 $$\begin{aligned} F_{i,n}(S_{i,n})=\theta _{i,ns}^{*T}\varsigma _{i,ns}(S_{i,n})+\varepsilon _{i,ns}\le {\bar{\theta }}_{i,ns}^{*T}{\bar{\varsigma }}_{i,ns}(S_{i,n}) \end{aligned}$$

where $|\varepsilon _{i,ns}|\le \varepsilon _{i,ns}^*$ with $\varepsilon _{i,ns}^*>0$. ${\bar{\varsigma }}_{i,ns}^T(S_{i,n})=[\varsigma _{i,ns}^T(S_{i,n}),1]$ and ${\bar{\theta }}_{i,ns}^{*T}=[\theta _{i,ns}^{*T}, \varepsilon _{i,ns}^*]$. By Lemma 4 and noting that $0<\varsigma _{i,ms}^T(\cdot )\varsigma _{i,ms}(\cdot )\le 1$, one has

72 $$\begin{aligned} |z_{i,n}|^{1-\tau }(\theta _{i,ns}^{*T}\varsigma _{i,ns}(S_{i,n})+\varepsilon _{i,ns})&\le |z_{i,n}|^{1-\tau }\zeta _{i,n}^{1-\tau \over 2}\nonumber \\&\le \Im _i^{*{1-\tau \over 2}}|z_{i,n}|^{1-\tau }\nonumber \\&\le \chi z_{i,n}^2\Im ^{*}_i+{\bar{\Gamma }} \end{aligned}$$

where $$\zeta _{i,n}=(\sqrt{2}\Vert {\bar{\theta }}_{i,ns}^{*T}\Vert )^{2\over 1-\tau }$$.

Furthermore, one has

73 $$\begin{aligned} \dot{V}_{i,n}&\le -n_{1,1}|z_{i,1}|^{4\over 2-\tau }-n_{1,2}|z_{i,1}|^{8\over 2-\tau }+{\widetilde{\Im }}_i\left( \sum _{m=1}^{n-1}\chi z_{i,m}^2-\dot{{\hat{\Im }}}_i\right) -\sum _{m=2}^{n-1}(n_{m,2}-1)z_{i,m}^2-\sum _{m=2}^{n-1}n_{m,1}z_{i,m}^4\nonumber \\&\quad +{\textrm{sig}}^{2-\gamma _{n+1}}(z_{i,n})\big (u_i+k_n(z_{i,1}-{\hat{z}}_{i,1})\big )+\chi z_{i,n}^2\Im ^{*}+3\Gamma _{i,1}+{\bar{\mu }}_2\Vert e\Vert ^2+(n-1){\bar{\Gamma }} \end{aligned}$$

Then the actuator control protocol $u_i$ and adaptive law $\dot{{\hat{\Im }}}_i$ are derived as,

74 $$\begin{aligned} u_i&=-n_{n,1}{\textrm{sig}}^{2+\gamma _{n+1}}(z_{i,n})-n_{n,2}{\textrm{sig}}^{\gamma _{n+1}}(z_{i,n})-{\textrm{sig}}^{\gamma _{n+1}}(z_{i,n})\chi {\hat{\Im }}_i-k_n(z_{i,1}-{\hat{z}}_{i,1}) \end{aligned}$$

75 $$\begin{aligned} \dot{{\hat{\Im }}}_i&=\sum _{m=1}^n\chi z_{i,m}^2-\sigma {{\hat{\Im }}}_i \end{aligned}$$

Based on (74), we have,

76 $$\begin{aligned} \dot{V}_{i,n}&\le -n_{1,1}|z_{i,1}|^{4\over 2-\tau }-n_{1,2}|z_{i,1}|^{8\over 2-\tau }+{\widetilde{\Im }}_i\left( \sum _{m=1}^{n}\chi z_{i,m}^2-\dot{{\hat{\Im }}}_i\right) -\sum _{m=2}^{n}(n_{m,2}-1)z_{i,m}^2\nonumber \\&\quad -\sum _{m=2}^{n}n_{m,1}z_{i,m}^4+3\Gamma _{i,1}+{\bar{\mu }}_2\Vert e\Vert ^2+(n-1){\bar{\Gamma }} \end{aligned}$$

Substituting the adaptive law (75) into (76) yields,

77 $$\begin{aligned} \dot{V}_{i,n} \le -n_{1,1}|z_{i,1}|^{4\over 2-\tau }-n_{1,2}|z_{i,1}|^{8\over 2-\tau }+\sigma {\widetilde{\Im }}_i{{\hat{\Im }}}_i-\sum _{m=2}^{n}(n_{m,2}-1)z_{i,m}^2-\sum _{m=2}^{n}n_{m,1}z_{i,m}^4+(n-1){\bar{\Gamma }}+3\Gamma _{i,1}+{\bar{\mu }}_2\Vert e\Vert ^2 \end{aligned}$$

#### Remark 4

According to the property $0<\varsigma _{i,ms}^T(\cdot )\varsigma _{i,ms}(\cdot )\le 1$ and maximal norm estimation method, only an adaptive parameter ${{\hat{\Im }}}_i$ is introduced to design the controller in place of utilizing weight parameter vectors, which efficiently simplifies the algorithm and reduces calculation load.

## Stability analysis

The focus of this section is to analyze the stability of the controlled nonlinear MASs (3) under the derived observer-based containment protocol.

### Theorem 1

For the uncertain nonlinear MASs (3) with Assumption 1-3, the issue of containment control can be effectively addressed when the state observer is constructed as (15), the virtual controllers are chosen as (40), (53), and (66), an actuator control scheme with adaptive updating law (75) design as (74). Furthermore, the derived controller can ensure that the controlled systems are practical fixed-time stable and all followers are able to converge to the convex hull formed by leaders within fixed-time.

### Proof

Note that,

78 $$\begin{aligned} \varpi _{i,m}=\int _{v_{i,m}}^{{\hat{x}}_{i,m}}{\textrm{sig}}^{2-\gamma _{m+1}}\big ({\textrm{sig}}^{1/\gamma _m}(s)-{\textrm{sig}}^{1/ \gamma _m}({v_{i,m}})\big )ds\le 2|z_{i,m}|^{2-\tau } \end{aligned}$$

Choosing $0<\alpha ={2\over 2-\tau }<1$, $1<\beta ={4\over 2-\tau }$, one then has

79 $$\begin{aligned} \dot{V}_{i,n}&\le -n_{1,1}(z_{i,1}^2)^\alpha -n_{1,2}(z_{i,1}^2)^\beta -\sigma {\widetilde{\Im }}_i{{\hat{\Im }}}_i-\sum _{m=2}^{n}(n_{m,2}-1)\varpi _{i,m}^\alpha +{\bar{\mu }}_2\Vert e\Vert ^2\nonumber \\&\quad -\sum _{m=2}^{n}n_{m,1}\varpi _{i,m}^\beta +(n-1){\bar{\Gamma }}+3\Gamma _{i,1} \end{aligned}$$

Invoking (23), (30), (55) and (68), we select the overall Lyapunov function as

80 $$\begin{aligned} V=V_o+\sum _{i=1}^N\dot{V}_{i,n} \end{aligned}$$

Invoking (26) and (79), the form of $\dot{V}$ is given,

81 $$\begin{aligned} \dot{V}v&\le -{\bar{\lambda }}_1\Vert e\Vert ^2-\sum _{i=1}^Nn_{1,2}(z_{i,1}^2)^\beta -\sum _{i=1}^Nn_{1,1}(z_{i,1}^2)^\alpha -\sum _{i=1}^N\sum _{m=2}^{n}(n_{m,2}-1)\varpi _{i,m}^\alpha -\sum _{i=1}^N\sum _{m=2}^{n}n_{m,1}\varpi _{i,m}^\beta \nonumber \\&\quad +N(n-1){\bar{\Gamma }}-\sigma \sum _{i=1}^N{\widetilde{\Im }}_i{{\hat{\Im }}}_i+{Nn\over 2}\Vert \Theta ^{*}\Vert ^2+{1\over 2}\Vert \varepsilon \Vert ^2+\sum _{i=1}^N3\Gamma _{i,1} \end{aligned}$$

where$${\bar{\lambda }}_1=\lambda _1-{\bar{\mu }}_2>0$$.

For $\sigma {\widetilde{\Im }}_i{{\hat{\Im }}}_i$, we can obtain,

82 $$\begin{aligned} \sigma {\widetilde{\Im }}_i{{\hat{\Im }}}_i\le -{\sigma \over 2}{\widetilde{\Im }}_i^2+{\sigma \over 2}\Im _i^{*2} \end{aligned}$$

Based on Refs.^42,49^, it is known that for a bounded function ${\widetilde{\Im }}$ with constant $\Delta _\Im$ as the boundary, $0<\alpha <1$ and $\beta >1$ such that,

83 $$\begin{aligned} -{\sigma \over 2}{\widetilde{\Im }}_i^2\le -\left( {\sigma \over 4}{\widetilde{\Im }}_i^2\right) ^\alpha -\left( {\sigma \over 4}{\widetilde{\Im }}_i^2\right) ^\beta +\omega _\Im \end{aligned}$$

where $$\omega _\Im =(1-\alpha )\alpha ^{\alpha /1-\alpha }+({\Delta _\Im ^2\sigma /4})^\beta $$

Similar to (83), we can obtain,

84 $$\begin{aligned} -{\bar{\lambda }}_1\Vert e\Vert ^2\le -\left( {{\bar{\lambda }}_1\over 2}\Vert e\Vert ^2\right) ^\beta -\left( {{\bar{\lambda }}_1\over 2}\Vert e\Vert ^2\right) ^\alpha +\omega _e \end{aligned}$$

where $$\omega _e=(1-\alpha )\alpha ^{\alpha /1-\alpha }+({\bar{\lambda }}_1{\bar{e}}^2/2)^\beta $$.

Employing (83) and (84), yields

85 $$\begin{aligned} \dot{V}&\le -\eta _1V^a+\sum _{i=1}^Nn_{1,2}(z_{i,1}^2)^\beta -\sum _{i=1}^N\sum _{m=2}^{n}n_{m,1}\varpi _{i,m}^\beta +N(n-1){\bar{\Gamma }}+\sum _{i=1}^N3\Gamma _{i,1}\nonumber \\&\quad +{Nn\over 2}\Vert \Theta ^{*}\Vert ^2-\sum _{i=1}^N\left( {\sigma \over 4}{\widetilde{\Im }}_i^2\right) ^\beta -\big ({\bar{\lambda }}_1\Vert e\Vert ^2/2\big )^\beta +{1\over 2}\Vert \varepsilon \Vert ^2 -\sum _{i=1}^N\left( {\sigma \over 4}{\widetilde{\Im }}_i^2\right) ^\beta +\omega _\Im +\omega _e+\sum _{i=1}^N{\sigma \over 2}\Im _i^{*2}\nonumber \\&\le -\eta _1V^a-{\eta _2\over 2^{\beta -1}}V^\beta +\aleph \end{aligned}$$

where $$\eta _1={\textrm{min}}\{\sigma ^\alpha ,n_{1{{\textrm{min}}}},2^\alpha {\bar{\lambda }}_1^\alpha ,\}$$, $$n_{1{{\textrm{min}}}}={\textrm{min}}\{{n_{m,2}-1},n_{1,1}\}$$, $$\eta _2={\textrm{min}}\{({\sigma /2})^\beta ,n_{2{{\textrm{min}}}},{\bar{\lambda }}_1^\beta \}$$, $$n_{2{{\textrm{min}}}}={\textrm{min}}\{n_{m,1},n_{1,2}\}$$, $$\aleph =N(n-1){\bar{\Gamma }}+\sum _{i=1}^N3\Gamma _{i,1}+{Nn\over 2}\Vert \Theta ^{*}\Vert ^2+{1\over 2}\Vert \varepsilon \Vert ^2+\omega _\Im +\omega _e+\sum _{i=1}^N{\sigma \over 2}\Im _i^{*2}$$.

According to Lemma 5, we see that the solutions of nonlinear MASs (3) are practical fixed-time stable under the derived observer-based containment control protocol. Moreover, we have

86 $$\begin{aligned} \Psi =\left\{ {\mathop {\textrm{lim}}_{\textrm{t}\rightarrow \textrm{T}_{\textrm{s}}}}z_i|V_n\le {\textrm{min}}\left\{ \eta _1^{-{1\over \alpha }}\left( {\aleph \over 1-\eta _0}\right) ^{1\over \alpha }, \left( {\eta _2\over 2^{\beta -1}}\right) ^{-{1\over \beta }}\left( {\aleph \over 1-\eta _0}\right) ^{1\over \beta }\right\} \right\} \end{aligned}$$

in fixed-time, that is $\Vert z_i\Vert \le \Delta _\Psi$ in fixed time, where $\Delta _\Psi ={\textrm{min}}\big \{ \eta _1^{-{1\over \alpha }}\big ({\aleph \over 1-\eta _0}\big )^{1\over \alpha }, \big ({\eta _2\over 2^{\beta -1}}\big )^{-{1\over \beta }}\big ({\aleph \over 1-\eta _0}\big )^{1\over \beta }\big \}$, $0<\eta _0<1$. Invoking $z_{i,1}=\sum _{j=1}^N\alpha _{ij}(y_i-y_j)+\sum _{j=N+1}^{M+N}\alpha _{ij}(y_i-y_{jr})$, it is a fact that $z_1=\mathfrak {T}_1y+\mathfrak {T}_2y_r$, where $y=[y_1,\dots ,y_N]^T$, $y_r=[y_{({1+N})r},\dots ,y_{{(N+M)}r}]^T$ $z_1=[z_{1,1},\dots ,z_{N,1}]^T$. Furthermore, we have $y-(-\mathfrak {T}_1^{-1}\mathfrak {T}_2y_r)=\mathfrak {T}_1^{-1}z_1$. As the convex hull is created by the leader agents, which can be defined as $y_d(t)=-\mathfrak {T}_1^{-1}\mathfrak {T}_2y_r$, when $\forall t\ge T$, we have

87 $$\begin{aligned} \Vert y-y_d(t)\Vert \le {\Vert z_1\Vert \over \lambda _{\textrm{min}}(\mathfrak {T}_1)}={\Delta _\Psi \over \lambda _{\textrm{min}}(\mathfrak {T}_1)} \end{aligned}$$

**Figure 1:**
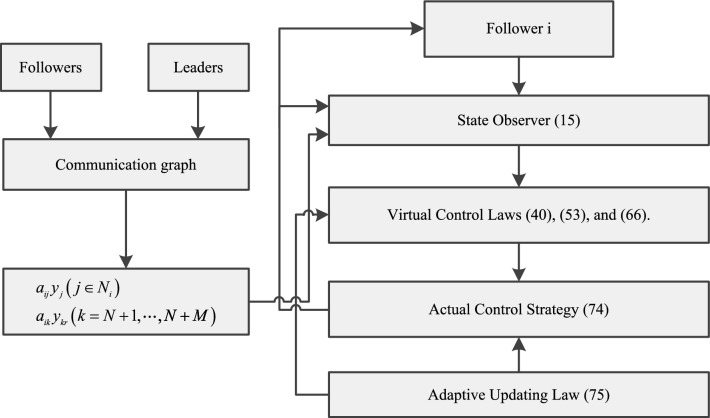
Block diagram of the fixed-time controller.

Therefore, it can conclude that the followers can realize the containment performance with a bounded containment error in fixed-time. The block diagram of the derived controller is given in Fig. 1. $\square$

### Remark 5

When using terminal sliding mode control methods to design fixed-time controllers, the nonlinear function in the terminal sliding mode controller contains fractional power terms, which may cause singularity problems when controlling second-order or higher-order systems. Although non-singular terminal sliding mode control methods can avoid singularity to some extent, they cannot guarantee non-singularity in the entire two-dimensional space and are only applicable to second-order systems, unable to be extended to higher-order systems. The proposed adding one power integrator method has effectively solved the singularity problem by introducing a class of power-term-integral items into the design of the virtual controller, effectively avoiding the possible singularity that may occur during differentiation. Based on this, a non-singular fixed-time controller based on power integral technology is proposed in this paper.

## Simulation examples

This section includes two simulation examples that test the validity of the derived controller.

**Figure 2:**
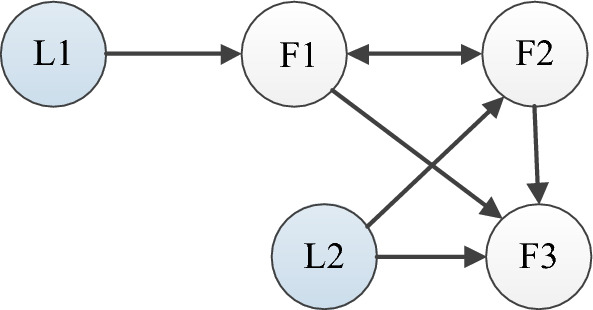
Directed communication graph.

### Example 1

The chosen example is composed by three followers and two leaders, which the communication graph can be given by Fig. 2. $F_1$, $F_2$, and $F_3$ represent followers 1-3, and $L_1$ and $L_2$ denote leaders 1–2 in Fig. 2. The dynamics of followers $i=1,2,3$ are defined as,

88 $$\begin{aligned} \dot{x}_{i,1}&=x_{i,2}+0.12x_{i,1}\textrm{sin}(x_{i,1})\nonumber \\ \dot{x}_{i,2}&=0.8\textrm{sin}(x_{i,1}x_{i,2})+u_i\nonumber \\ y_i&=x_{i,1} \end{aligned}$$

The dynamics of the leaders are,

89 $$\begin{aligned} y_{1r}&=0.6+0.3\textrm{sin}(0.15t)\nonumber \\ y_{2r}&=-0.3+\textrm{exp}(-t). \end{aligned}$$

The initial values are given as ${\hat{x}}_{1,1}(0)=0.1$, $x_{1,1}(0)=0.3$, ${\hat{x}}_{1,2}(0)=0.25$, $x_{1,2}(0)=1$, ${\hat{x}}_{2,1}(0)=0.1$, $x_{2,1}(0)=0.4$, ${\hat{x}}_{2,2}(0)=-0.15$, $x_{2,2}(0)=-1$, ${\hat{x}}_{3,1}(0)=0.1$, $x_{3,1}(0)=0.5$, ${\hat{x}}_{3,2}(0)=-0.25$, $x_{3,2}(0)=-1$, ${{\hat{\Im }}}(0)=[0.3,0.1,0.1]$. The design parameters are given as $\alpha _{12}=1$, $\alpha _{14}=1$, $\alpha _{13}=0$, $\alpha _{21}=1$, $\alpha _{15}=0$, $\alpha _{24}=0$, $\alpha _{23}=0$, $\alpha _{25}=1$, $\alpha _{31}=0.9$, $\alpha _{32}=0.2$, $\alpha _{34}=0$, $\alpha _{35}=1.3$, $n_{1,1}=2$, $n_{2,1}=2$, $n_{1,2}=2$, $n_{2,2}=2$, $k_{1}=15$, $k_{2}=85$, $\chi _i=0.2$, $\sigma =0.1$, ${\bar{\mu }}_{i,1}=2$, $\tau =-1/8$.

**Figure 3:**
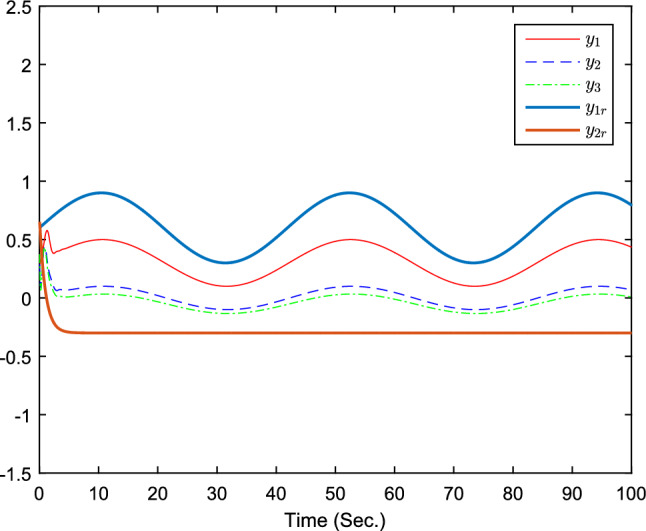
The curves of $y_i(i=1,2,3)$ and $y_{jr}(j=1,2)$.

**Figure 4:**
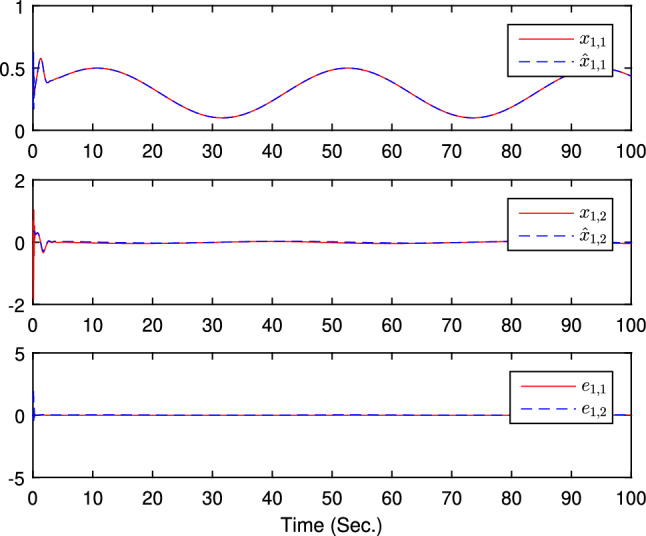
The curves of state $x_{1,j}$, ${\hat{x}}_{1,j}$, and observer error $e_{1,j}(j=1,2)$.

**Figure 5:**
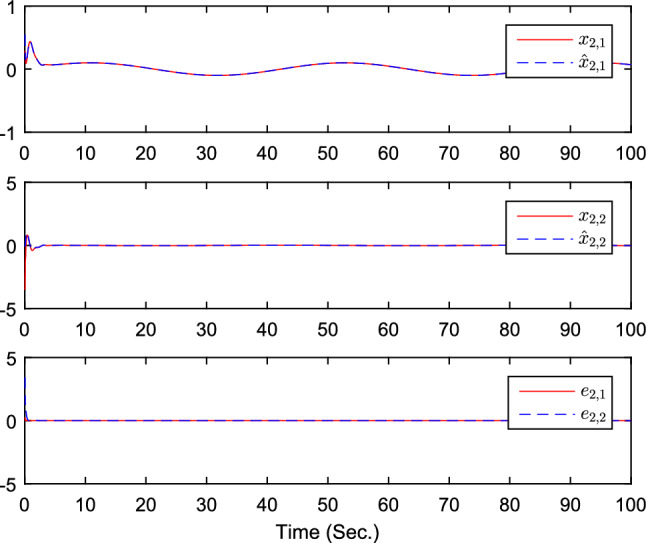
The curves of state $x_{2,j}$, ${\hat{x}}_{2,j}$, and observer error $e_{2,j}(j=1,2)$.

**Figure 6:**
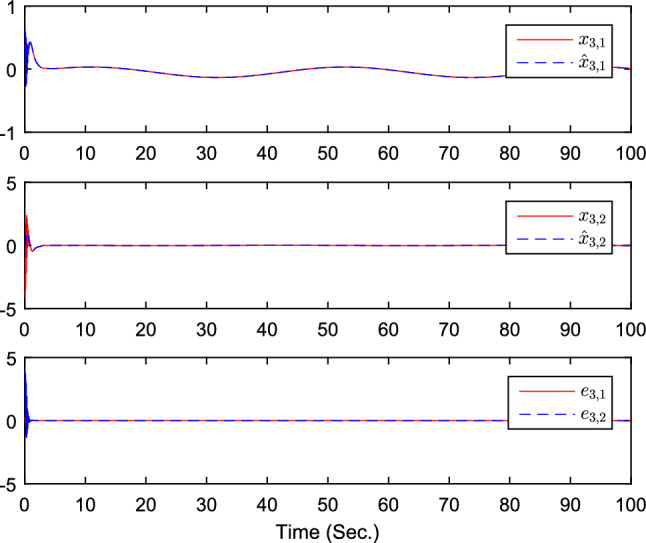
The curves of state $x_{3,j}$, ${\hat{x}}_{3,j}$, and observer error $e_{3,j}(j=1,2)$.

**Figure 7:**
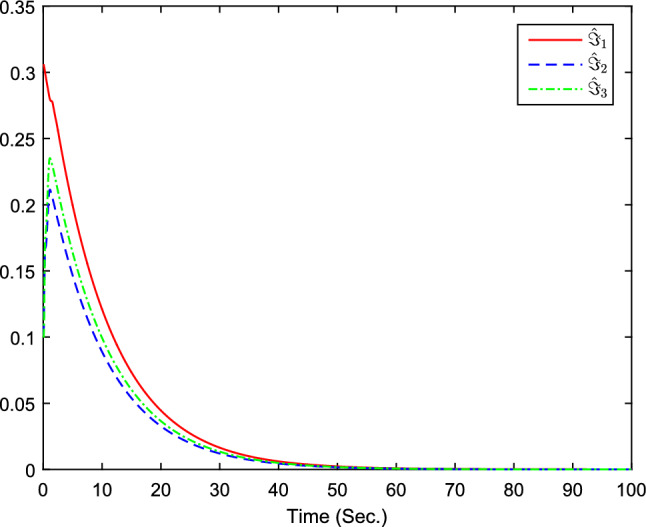
The curves of ${\hat{\Im }}_i(i=1,2,3)$.

**Figure 8:**
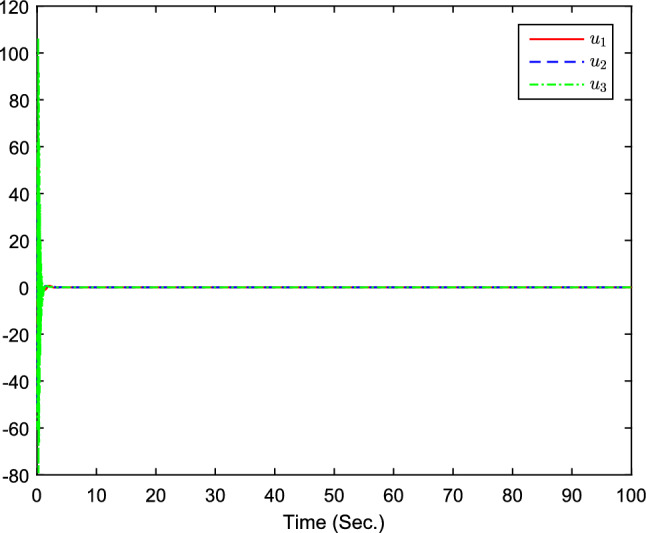
The curves of $u_i(i=1,2,3)$.

The simulation results are exhibited by Figs. 3, 4, 5, 6, 7 and 8. Figure 3 shows the curves of ${y_i}$, $y_{1r}$ and $y_{2r}$. Figures 4, 5 and 6 show the curves of the state estimation values. From Figs. 7 and 8, the curves of ${\hat{\Im }}_i$ and $u_i$ are shown, respectively.

### Example 2

The communication topology in this scenario remains the same as in Example 1. Each follower agent represents a damped car system. The dynamics of such a system can be represented by the following equation

90 $$\begin{aligned} \dot{x}_{i,1}&=x_{i,2}+f_{i,1}(x_{i})\nonumber \\ \dot{x}_{i,2}&={1\over M}(-k_0e^{-x_{i,1}}x_{i,1}-F_dx_{i,2}+u_i)\nonumber \\ y_i&=x_1 \end{aligned}$$

where $f_{i,1}(x_{i})=-0.01{\textrm{cos}}(x_{i,1})\textrm{sin}(x_{i,2})$ is the external disturbances, $x_{i,1}$ is the displacement of the car with respect to the rest position, and $x_{i,2}$ is the speed of the car. $F_d=1.1$ Ns/m is the damping factor. $M=1$kg is the mass of the car, and $K=k_0e^{-x_{i,1}}$ denotes the stiffness of the spring, $k_0=0.33$N/m. The models of the leaders are

91 $$\begin{aligned} y_{1r}&=0.7+0.4\textrm{sin}(0.1t)\nonumber \\ y_{2r}&=-0.3+0.4\textrm{sin}(0.1t). \end{aligned}$$

**Figure 9:**
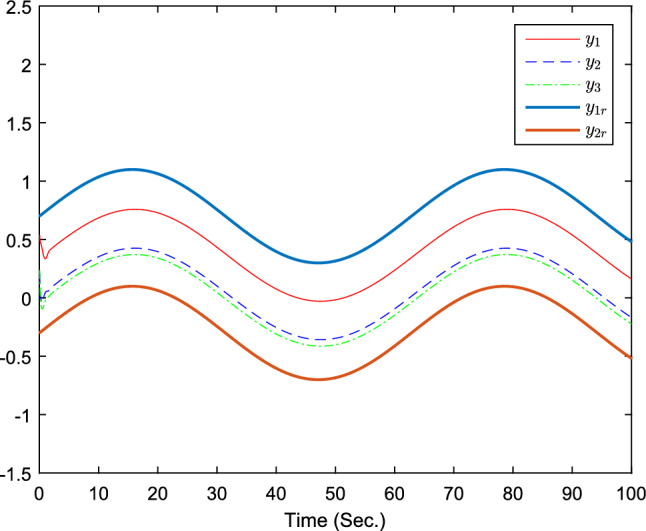
The curves of $y_i(i=1,2,3)$ and $y_{jr}(j=1,2)$.

**Figure 10:**
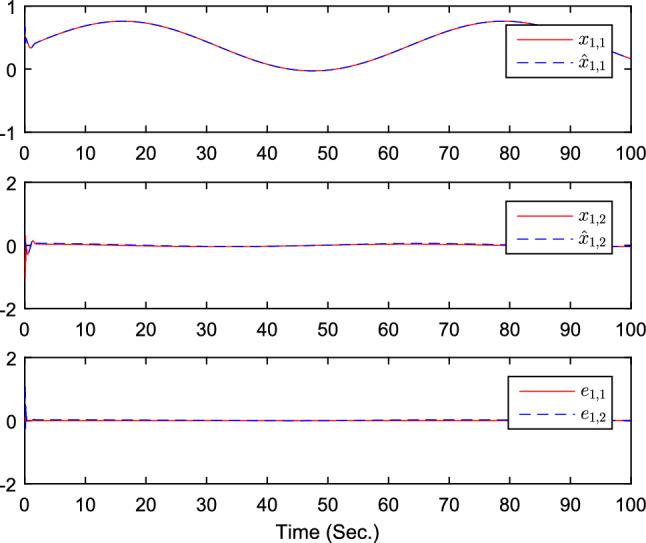
The curves of state $x_{1,j}$, ${\hat{x}}_{1,j}$, and observer error $e_{1,j}(j=1,2)$.

**Figure 11:**
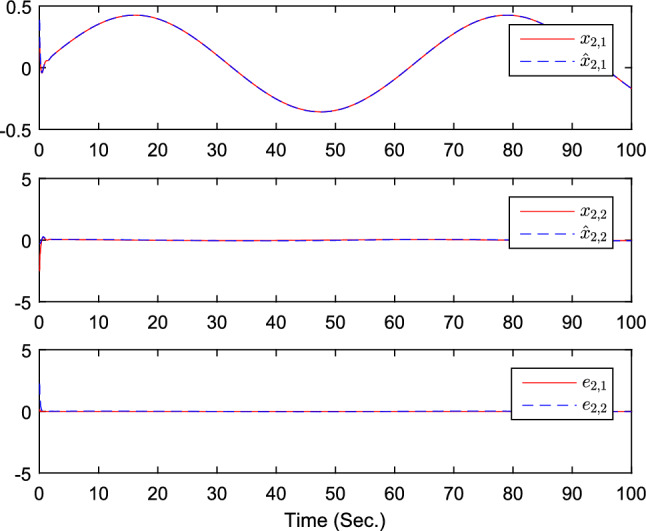
The curves of state $x_{2,j}$, ${\hat{x}}_{2,j}$, and observer error $e_{2,j}(j=1,2)$.

**Figure 12:**
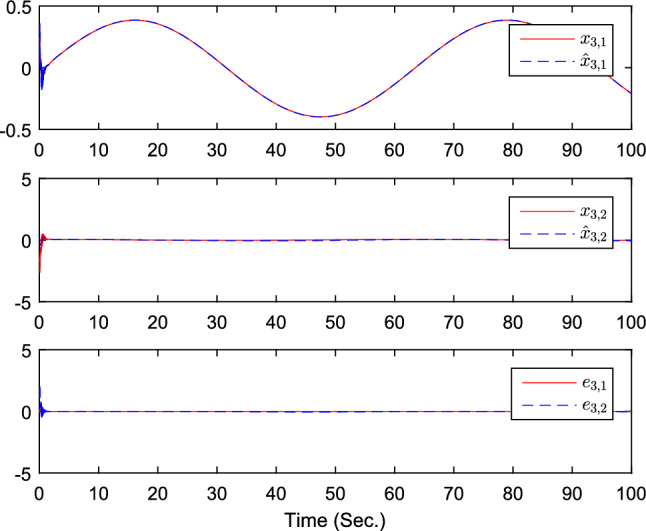
The curves of state $x_{3,j}$, ${\hat{x}}_{3,j}$, and observer error $e_{3,j}(j=1,2)$.

**Figure 13:**
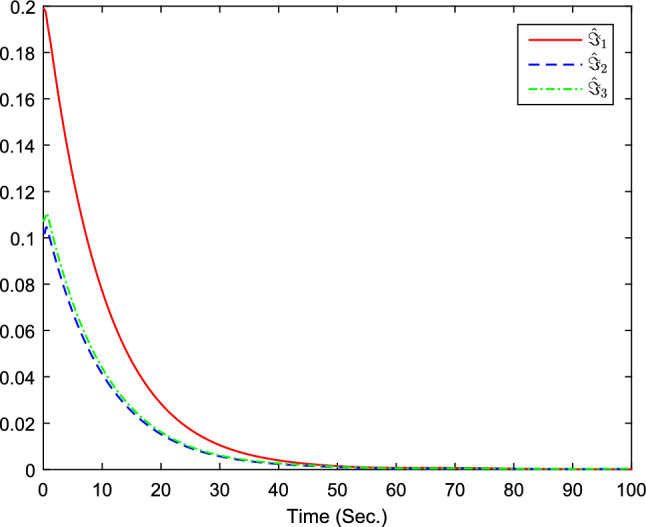
The curves of ${\hat{\Im }}_i(i=1,2,3)$.

**Figure 14:**
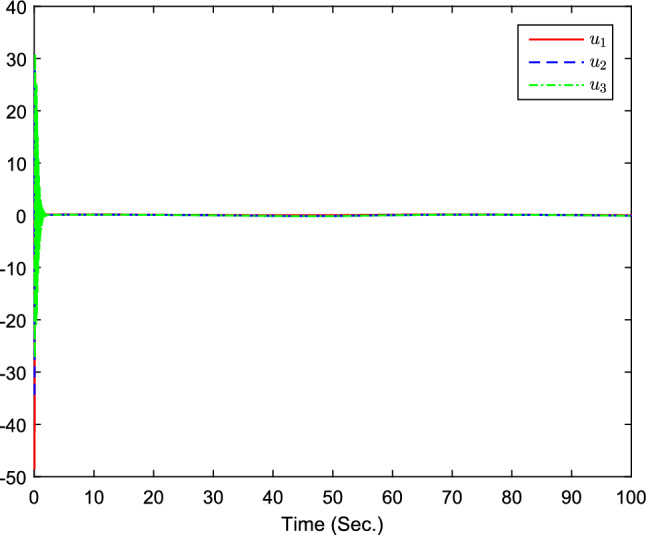
The curves of $u_i(i=1,2,3)$.

The initial values are given as ${\hat{x}}_{1,1}(0)=0.1$, $x_{1,1}(0)=0.5$, ${\hat{x}}_{1,2}(0)=0.2$, $x_{1,2}(0)=2$, ${\hat{x}}_{2,1}(0)=0.1$, $x_{2,1}(0)=0.3$, ${\hat{x}}_{2,2}(0)=-0.15$, $x_{2,2}(0)=-1$, ${\hat{x}}_{3,1}(0)=0.1$, $x_{3,1}(0)=0.3$, ${\hat{x}}_{3,2}(0)=-0.15$, $x_{3,2}(0)=-1$, ${{\hat{\Im }}}(0)=[0.2,0.1,0.1]^T$. The design parameters are given as $\alpha _{12}=1$, $\alpha _{21}=1$, $\alpha _{31}=0.9$, $\alpha _{13}=0$, $\alpha _{23}=0$, $\alpha _{14}=1$, $\alpha _{32}=0.2$, $\alpha _{24}=0$, $\alpha _{15}=0$, $\alpha _{25}=1$, $\alpha _{34}=0$, $\alpha _{35}=1.3$, $n_{1,1}=1.5$, $n_{1,2}=2$, $n_{2,1}=1.5$, $n_{2,2}=2$, $k_{1}=15$, $k_{2}=80$, $\chi =0.1$, $\sigma =0.1$, ${\bar{\mu }}_{i,1}=2$, $\tau =-1/5$. Figure 9 is the containment control result, and we see that all follower agents’ outputs are able to converge to the convex hull formed by the all leaders’ outputs $y_{ir}$ . Figures 10, 11 and  12 show the curves of the state estimation values, which prove the validity of the constructed state observer. From Fig. 13, the curves of ${\hat{\Im }}_i$ are shown. The curves of $u_i$ can be obtained from Fig. 14. The control protocol (74) that has been derived has been proven to be effective in achieving ideal containment control performance.

Based on above results, the derived observer-based fixed-time containment controller confirms the practical fixed-time stability of the controlled every follower agent. Besides, it is noticeable that the outputs $y_i$ are able to converge into the convex hull formed by the leader agents in fixed-time.

### Remark 6

A new control strategy, which involves designing a state observer and implementing fixed-time containment control, is presented for nonlinear multiagent systems in this paper. In contrast to the current finite-time controllers, our derived containment control protocol can confirm the controlled systems are practical fixed-time stable, in which its settling time of the controlled systems can be determined without the initial states. As the states of each follower cannot be directly measured, an observer-based fixed-time containment control protocol is formulated, which can confirm the controlled multiagent systems are practical fixed-time stable and the observer errors are bounded within fixed-time. In comparison with recent results on fixed-time control by using sliding mode control approach, the developed fixed-time containment control protocol via the adaptive backstepping and adding one power integrator approach in this article can avoid the singular phenomenon. Moreover, the developed fixed-time controller denotes a new attempt for MAS systems subject to unmeasurable states and unknown nonlinear functions by backstepping approach and adding one power integrator approach, which can prevent the singular and non-continuous phenomenon.

## Conclusion

The focus of this article was to study the problem of fixed-time containment control in MASs that have unmeasurable states and unknown nonlinear functions. To address the issue of unmeasurable states, a new state observer was constructed. A fixed-time adaptive containment control algorithm was formulated by integrating the adding one power integrator technique and backstepping approach. The proposed algorithm effectively resolves the singular problem in this paper. It was proved that the solutions of the controlled system are practical fixed-time stable under the formulated observer-based containment fixed-time controller, and the follower agents are able to converge to the convex hull formed by the leaders in fixed-time. Finally, simulation results tested the validity of the developed fixed-time adaptive control protocol. Future study will concentrate on formulating a fixed-time containment controller for MAS that involve switching topologies and mismatched disturbances .

## Data Availability

All data generated or analysed during this study are included in this published article.
